# “The inverse equality hypothesis” and “cultural consonance”: two Brazilian contributions for a new public health agenda

**DOI:** 10.1590/S1516-31802001000100001

**Published:** 2001-01-02

Social determinants of health are an old public health issue. However, for many decades, the focus of epidemiology was more centered on biological determinants. At first, during the predominance of infectious diseases like cholera, smallpox, and tuberculosis, the most relevant aspects of the causal chain were the agents: viruses, bacteria, and protozoa. At the beginning of the 20th century, microbiology was described using a mixed of hope and hype, in the same way that today molecular biology and genetics are presented by the lay press as “revolutionary”, “the future”, and other similar terms.

The discovery of the microbial agents of diseases built up a strong belief that the eradication of all infectious diseases would become possible. Nevertheless, the knowledge that cholera is a water-borne disease (described by the seminal work of John Snow analyzing water sources in London in the 18th century) preceded Robert Koch's description of the bacterium Vibrio cholerae by more than one century.

The epidemiological transition observed between World Wars I and II in Europe and the United States, and in Brazil during the 1960's, i.e. the predominance of cardiovascular diseases and cancer over infectious diseases, changed the paradigm from being microbial to a new one based on risk factors. Studies using cohort designs such as the Framingham Heart Study, and case-control studies such as the smoking and lung cancer studies by Doll & Hill in the United Kingdom brought in new risk factors like smoking, high blood pressure and high cholesterol. However, the microbial and risk factor paradigms are very restricted in explaining the causes of diseases, and especially in constructing “hierarchical causal chains” for diseases.

One corollary of both theories is the prospect of the “elimination” of viruses (like the smallpox virus) or lifestyles (like the smoking habit). Obviously, the extinction of the smallpox virus (if this has really been possible) is closely related to the extinction of the smallpox disease. Likewise, a reduction in the number of smokers will decrease the burden of lung cancer. However, “elimination” as proposed for public health agents is as ingenuous as it is attractive. It is more an adaptation of military jargon than a public health concept based on epidemiology.

In Brazil, during the 1980's, some faculties of medicine, especially those with Departments of Social Medicine and Schools of Public Health, developed an alternative theory to face up to the “biological reductionism” that has been termed “social epidemiology”. In fact, they developed more essays on epistemology than new theories or methods on epidemiology. In contrast, two Brazilian teams working on empirical data collected in field surveys in Ribeirão Preto (State of Sao Paulo) and Pelotas (State of Rio Grande do Sul) constructed two new concepts that will be very useful in understanding the determinants of health abroad.

In Pelotas, Southern Brazil, at the beginning of the 1980's, F.C. Barros and C.G. Victora started a cohort of newborns over a one-year period that was analyzed and published in a seminal book.^1^ Now, these authors and colleagues are proposing a new theory about the impact of public health interventions.^2^ They have compared the Pelotas birth cohort with mortality rates in the Americas and resolute governmental action that has been taking place to decrease infant mortality in the rural areas of the State of Ceará, one of the poorest states in Brazil. They hypothesized (and, moreover, ascertained) that “inequality ratios tend to decrease only when the wealthy have reached a new minimum achievable level of morbidity or mortality for those public-health interventions.” The comprehension of this problem is very important for everybody working towards changing the dramatic differences in health indicators within a country or between countries.

In Ribeirão Preto, Sao Paulo State, a collaboration covering more than a decade between J. E. Santos of the Ribeirão Preto Medical School and William Dressler of the University of Alabama has been bringing to light the high burden of hypertension in Brazil.^3^ They have co-authored more than a dozen original articles about lifestyles and blood pressure among workers in Ribeirão Preto. Their latest paper describes “a complementary model of social and cultural influences on disease risk, placing greater emphasis on how individuals are able to approximate, in their own behaviors, shared cultural models of life, referred to as ‘cultural consonance’”. The authors proved that “the higher an individual's cultural consonance, the lower his or her blood pressure. These results indicate the importance of linking different levels of analysis - the cultural, the individual, and the biological - to understand disease risk.”

Recently, the Harvard School of Public Health and the London School of Hygiene and Tropical Diseases published textbooks emphasizing the social determinants of health.^4,5^ This is a new issue for them, but not for us. Another significant news item occurred some months ago, when “Nature” published work by Brazilian scientists on genome research surpassing that of well-funded American and European laboratories. Thus, it will be possible for Brazilian epidemiologists to be able to reach the same level of relevance as obtained by their colleagues within genome research. They need the same support and cohesion shown by scientist colleagues at the bench.
